# Tai Chi versus routine exercise in patients with early- or mild-stage Parkinson's disease: a retrospective cohort analysis

**DOI:** 10.1590/1414-431X20199171

**Published:** 2020-02-10

**Authors:** Quanhao Li, Jinmei Liu, Fei Dai, Fengling Dai

**Affiliations:** 1 Department of Neurology, Gaomi People's Hospital, Gaomi, Shandong, China; 2 Department of Neurosurgery, Gaomi People's Hospital, Gaomi, Shandong, China; 3 Department of Gastroenterology, Lishui People's Hospital, Lishui, Zhejiang, China

**Keywords:** Exercise, Fall, Levodopa, Parkinson's disease, Physical therapy, Tai Chi

## Abstract

Parkinson's disease cannot be cured but symptoms can be improved by making use of physical therapy. The objective of the study was to compare the effect of routine exercises and Tai Chi on physical and clinical performance in elderly people suffering from Parkinson's disease. Data from interviews, physical and clinical performance, and levodopa consumption of 500 patients with confirmed Parkinson's disease (severity level I to III) were collected and analyzed. Participants who received 80 min/day Tai Chi 3 times/week for 2 months were included in the Tai Chi (TC) group (n=250) and those who received 90 min/day routine exercise 3 times/week for 2 months were included in routine exercise (RE) group (n=250). Timed up-and-go, 50-foot speed walk, and functional reach were improved by Tai Chi and routine exercise (P<0.05 for all) but intensities of Tai Chi for improvement of such parameters was higher than routine exercise. Incidence of falls was decreased by both physical therapies (P<0.05 for all) but more for the TC group (P<0.0001, q=38.512). In the TC group, at the end of follow-up, 22 (9%) patients were successful in withdrawal of levodopa treatment. Also, the dose of levodopa was decreased in patients of the TC group who had to continue levodopa. Tai Chi had the potential to slow down the progression of symptoms of Parkinson's disease and delayed the introduction of levodopa (level of evidence: III).

## Introduction

Parkinson's disease (PD) is a neurodegenerative disorder that is highly prevalent, complex, and progressive. It is a disorder of the nervous system that severely impairs the motor role of the body. As the disease advances, motor and non-motor signs and symptoms get more observable (1). In some patients, problems related to thought and behavior also occur at an advanced stage of the disease. As the disease advances, dementia, anxiety, and depression become common. Other symptoms that appear in PD patients are sleeping and emotional disturbances. All these symptoms come collectively under one umbrella term: Parkinsonism (2).

PD is the second most prevalent neurodegenerative complex disorder and 4 million people are diagnosed with PD worldwide (1–2% of the world population). The disorder is dependent on the age of the patient and very few cases occur before the age of 50 years, and after the age of 60 years there is a severe rise in symptoms of the disease (3). It has affected more than 1% of the population older than 50 years (4). PD is more prevalent in males compared to females because the female-specific hormone estrogen has a neuroprotective effect that delays PD onset (5). The causative agent behind PD is still unknown but it is believed to be due to genetic and environmental factors including exposure to toxic chemicals, head trauma, dysfunction of the mitochondrion, and X-linked genetic factors. A reduced risk of PD in people who drink coffee and tea (6) has been reported. It is a challenging and complex disease because of its progressive nature. Impairments associated with gait and instability of posture are the hallmark of this disease. The person can also experience dementia (7). Despite medical advances, PD cannot be cured but symptoms can be improved by making use of several treatment options. PD patients place strains on society, finances, and health care (8).

Chronic symptoms of this disease lead patients to therapies that help alleviate the symptoms. One such treatment is physical therapy, which can be aerobic training, e.g., stationary bicycle and treadmill training (9), Tai Chi, a Chinese martial art that has been proven to enhance the physical and motor capability of PD patients (10), dance, which helps with partner support, social engagement, balance, and strength (11), and agility training, a sensorimotor agility program (12).

Physiotherapy has introduced non-pharmacological treatment options including conventional exercise and physical therapy. Both of these terms are often confused and used as synonyms (13). The American College of Sports Medicine has defined exercise as a sub-class that comes under the more general category of physical activity that involves structured and planned body movements to maintain or improve physical activity, whereas physical therapy is any activity performed via contraction of skeletal muscles and involves the expenditure of energy (14).

Tai Chi is an exercise based on balance maintenance guided by the yin-yang theory of traditional Chinese medicine originating 6000 years ago. Tai Chi is a safe and effective technique that helps the body and brain, and consists of approximately 108 intricate exercise steps (15) and 4 basic steps. Dao Yin consists of gentle stretches that help the body warm up; Chi Kung exercise consists of synchronized breathing to help maintain the body's balance, posture, and reduce stress; Tai Chi Chuan is a series of movements performed in a slow, flowing, and relaxed manner that spans over 20 min; and push hand practice, which is only recommended for certain cases where the participant wants to learn the philosophy of Tai Chi.

Progressive resistance exercise can affect the balance maintenance in PD patients effectively (16,17). The National Parkinson Foundation has recommended Tai Chi for PD patients as complementary therapy but little evidence has been provided via clinical trials.

The primary objective of the study was to compare and analyze the effect of routine exercises and Tai Chi on physical and clinical performance of elderly people suffering from PD. The secondary objective was to test the hypothesis that Tai Chi delays the introduction of levodopa.

## Material and Methods

### Ethics approval and consent to participate

The original protocol (GPH/CL/15/2016 dated 15 June 2019) of the study had been approved by the Gaomi People's Hospital review board. An informed consent form was signed by all participating patients or their relative(s) (legally authorized person) regarding non-treatment interventions, evaluations, and publications of the study in all formats irrespective of time and language. The study adhered to the law of China, the strengthening the reporting of observational studies in epidemiology (STROBE), cohort study statement, and the 2008 Helsinki Declaration.

### Inclusion and exclusion criteria

All patients aged 18 years and above with confirmed PD (diagnosed as per UK Brain Bank criteria (18)), severity level I to III (very early-stage, early-stage, and mild-stage), that could move independently, had no other severe neurological disorder, had not participated in any kind of physical therapy program in the previous 2 months, and did not have other severe orthopedic disorders were included in the analysis.

Patients with PD disease severity level IV (advanced-stage) and patients who had received any physical therapy exercise in the previous 2 months were excluded from the analysis.

### Cohorts

PD patients who received 80 min per day Tai Chi training 3 times a week for 2 months were included in the TC group and those who received 90 min per day routine exercise (treadmill training, aerobic training, and dance) 3 times a week for 2 months were included in RE group.

### Tai Chi program

Participants were instructed to perform 6 moves of Tai Chi that originated from the traditional Yang Tai Chi style. Six forms of moves were performed: wave hands like clouds, Taijiquan (part wild horse's mane), stepping up and then moving down, fists striking the ears of the opponent, repulse monkey, and grasping the peacock's tail. The exercise routine was to perform these six moves in a repetitive manner for 3 consecutive days in a week for two months.

### Routine exercise (RE) program

Participants performed routine exercises including treadmill training, aerobic training, and dance for 3 consecutive days in a week over a period of 2 months.

### Instructions to the class and monitoring

The investigators coached and supervised the training session for 2 months. Both investigators had more than 5 years of experience in physical therapy, cardiopulmonary resuscitation, and first aid. Participants were asked whether any discomfort was faced and their responses were recorded after the end of the training session. Changes were made on an individual basis in the program.

### Outcome measurements

The investigators carried out pre-assessment and post-assessment (after 2 months of intervention) to assess the improvement in motor function.

### Final interview

The final interview was conducted by the supervisors at the end of the 2-month trial period. Responses of the participants were recorded and summarized for both groups.

### Measurement of physical performance

Physical performance was measured by three tests: the 50-foot speed test, which estimates the time by the patient takes to walk a 50-foot distance; the timed up-and-go test, which evaluates the time required by a participant to stand up from a chair, walk 10 feet, and come back and sit down on the chair; the functional reach test, which measures 16 common functional activities, e.g. unloading groceries, making a bed, climbing 3 steps onto a platform with luggage, etc. (9) (0: minimum and 100: maximum). Incidence of falls was also recorded before intervention (previous 6-months) and within 6-months (from the start of physical therapy and follow-up period). Participants were helped and assisted to perform the tasks whenever needed.

### Clinical evaluations

Total number of patients who had to take levodopa or equivalent treatment and the dose per day were also noted before the intervention and within 6-months (from the start of treatment and follow-up period). Adverse events were also recorded.

### Statistical analysis

SPSS (IBM, USA) was used for statistical analysis. Chi-squared independent test was used for categorical data and paired sample *t*-test for continuous data. Tukey test (considering critical value [*q*]>3.314 as significant) was used for *post-hoc* analysis. The results were considered significant at P<0.05.

## Results

### Enrollment

**Figure 1:**
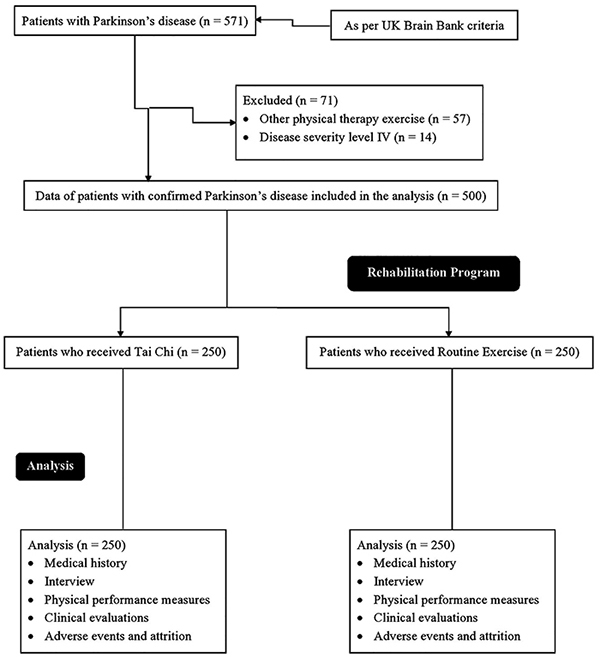
Flow diagram of the study.

From December 1, 2017 to January 15, 2019, a total of 571 patients were diagnosed with PD in the Department of Neurology of the Gaomi People's Hospital, Gaomi, Shandong, China and the Lishui People's Hospital, Lishui, Zhejiang, China. All patients were subjected to exercise during the rehabilitation program in the Department of Rehabilitation Sciences of the institutes. Among them, 57 patients were subjected to physical therapy other than Tai Chi or routine exercise (e.g., yoga, the art of living, etc.) and 14 patients had PD level IV. Therefore, data of these patients were excluded from the analysis. Data regarding interview and physical measurements of 500 patients with confirmed PD who received Tai Chi (n=250) or routine exercise (n=250) were included in the analysis (Figure 1).

### Medical history of patients

Among the enrolled PD patients, male to female ratio was 2.5:1. Some of the patients had started levodopa or equivalent treatment in the previous 6 months. The other demographical and clinical parameters are reported in Table 1. There were no significant differences for the parameters between both groups at enrollment (P>0.05 for all).

**Table 1:** Demographical, anthropological, and clinical parameters of the enrolled Parkinson's disease patients who performed Tai Chi (TC) or routine exercise (RE) programs.

Parameter	Total population (n=500)	Groups		P value
		TC (n=250)	RE (n=250)	
Gender				
Male	356 (71)	182 (73)	174 (70)	0.489
Female	144 (29)	68 (27)	76 (30)	0.489
Ethnicity				
Han Chinese	439 (88)	220 (88)	219 (88)	0.918
Mongolian	35 (7)	17 (7)	18 (7)	0.918
Tibetan	16 (3)	7 (3)	9 (3)	0.918
Taiwanese	6 (1)	4 (1)	2 (1)	0.918
North Korean refugee	4 (1)	2 (1)	2 (1)	0.918
Age (years)	61.21±8.4	62.52±5.45	61.89±4.55	0.161
Age at onset of PD (years)	56.56±5.78	56.12±4.55	55.85±5.12	0.533
Duration of disease (years)	5.12±3.52	4.99±2.52	5.35±3.01	0.148
Body mass index (kg/m^2^)	25.07±1.14	24.87±1.11	25.07±1.21	0.055
Parkinson's disease severity level	256 (51)	127 (51)	129 (52)	0.982
I	177 (35)	89 (36)	88 (35)	0.982
II	67 (14)	34 (13)	33 (13)	0.982
III				
Incidence of falls in last 6 months	7.52±2.15	7.45±1.18	7.65±1.26	0.068
Levodopa or equivalent treatment in last 6 months				
Patients were put on levodopa or equivalent treatment	112 (22)	54 (22)	58 (23)	0.748
Patients were not put on levodopa or equivalent treatment	388 (78)	196 (78)	192 (77)	0.748
Levodopa or equivalent dose (mg/day)	450±35	425±45	435±60	0.323
Timed up-and-go test (s)	17.01±2.22	17.09±2.31	16.82±3.52	0.311
50-foot walk test (s)	10.55±2.85	10.49±2.79	10.78±3.71	0.324
Functional reach (0: minimum and 100: maximum)	27.25±5.85	26.46±5.71	27.49±6.52	0.061

Categorical data are reported as number (percentage) and continuous data are reported as means±SD. Parkinson's disease severity level: I: very-early stage; II: early stage; and III: mild-stage. Chi-squared independent test was used for categorical data and paired sample *t*-test for continuous data. P<0.05 was considered significant.

### Final interview

In general, all the participants were happy with their exercise program and found exercise movements easy, safe, and appropriate. Participants reported that exercises helped them improve their confidence and balance. More patients in the RE group responded 'not sure' or 'no' in terms of stability during the final interview. The other results of the survey are summarized in Table 2.

**Table 2:** Response of Parkinson's disease (PD) patients who performed Tai Chi (TC) or routine exercise (RE) programs recorded in the final interview.

Questions	TC group (n=250)		RE group (n=250)		P value
	Yes (%)	No or not sure (%)	Yes (%)	No or not sure (%)	
Enjoyment during program	250 (100)	0 (0)	250 (100)	0 (0)	N/A
Easy to learn	245 (98)	5 (2)	250 (100)	0 (0)	0.07
Safe to perform	250 (100)^*^	0 (0)	240 (96)	10 (4)	0.004
Enhanced my confidence	250 (100)	0 (0)	250 (100)	0 (0)	N/A
I feel more balanced and stable	243 (97)^*^	7 (3)	230 (92)	20 (8)	0.018
Intention to continue	250 (100)	0 (0)	250 (100)	0 (0)	N/A
Understood instructions	243 (97)	7 (3)	245 (98)	5 (2)	0.77
Would recommend it to other PD patients	250 (100)	0 (0)	250 (100)	0 (0)	N/A

Data are reported as number (percentage). Chi-squared independent test was performed to statistical analysis. N/A: Not applicable.

*P<0.05 compared to RE group.

### Physical performance measures

Results for the timed up-and-go test, 50-foot walk test, and functional reach were improved by Tai Chi and routine exercise (P<0.05 for all) but the TC group had greater improvements (Supplementary Table S1).

### Clinical evaluations

In the TC group, at the end of follow-up, 22 (9%) patients were successful in withdrawing levodopa or equivalent treatment. Also, a decrease in the daily dose of levodopa or equivalent was seen in the TC group in those who continued on levodopa (Supplementary Table S2).

### Incidence of falls

**Figure 2:**
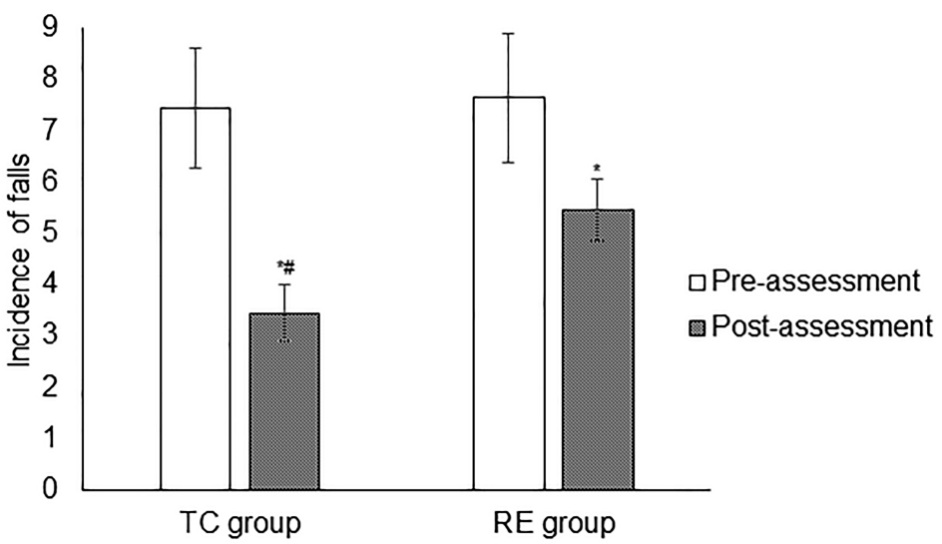
Incidence of falls for Parkinson's disease patients who performed Tai Chi (TC) or routine exercise (RE) programs. Data are reported as means±SD. Total number of patients enrolled were 250 for both groups. *P<0.05 compared to pre-assessment; ^#^P<0.05 compared to RE group. Paired sample *t*-test was performed for statistical analysis. Pre-assessment: Incidence of falls in previous 6-months. Post-assessment: Incidence of falls within 6-months from start of physical therapy and follow-up period.

Incidence of falls within 6 months from the start of treatment to the follow-up period were decreased in the TC group (7.45±1.18 *vs* 3.45±0.55, P<0.0001, q=60.463) and RE group (7.65±1.26 *vs* 5.46±0.59, P<0.0001, q=32.876), but Tai Chi decreased incidence of falls significantly more than routine exercise (P<0.0001, q=38.512, Figure 2).

### Adverse events and attrition

No attrition occurred during the whole program period or during follow-up. Also, no injury or fall was observed in patients of the TC group. Among the participants of the RE group, just 2 subjects experienced a fall during the first week of intervention.

## Discussion

### Patients' response towards Tai Chi and routine exercises

All the enrolled participants showed interest and enjoyed physical therapy. Participants of both groups reported satisfaction. Participants of both groups felt more balanced with better stability in their gait. The results of the study were consistent with a previous study (19). The program was safe and successful in terms of achieving the objective of making elderly PD patients familiar with Tai Chi and other exercises.

### Physical performance measures

The results of our study were consistent with randomized controlled trials (9,10). In early and mild-stage PD, when the development of disease starts, symptoms like shaking of hands, difficulty in walking, slowness in movement, and rigidity become obvious (1). Exercise (20) and dance (11) help PD patients in the improvement of both motor and non-motor complications. Tai Chi is a common Chinese martial art that involves posture maintenance and controlled movements in a slow and flowing fashion. This exercise technique includes movements such as extension of knees and hips, weight shifting and flexion, rotation of the trunk, and coordinated movement of arms (21). Treadmill training might be better suited for young and fit people than for elderly PD patients (22). The Tai Chi program together with proper medication resulted in improved motor function and mobility (23). Routine exercise does not change basic symptomatology (24). The study showed that routine exercises do not help in the enhancement of stability and Tai Chi does help in improving motor activity of PD patients.

### Incidence of falls

Tai Chi decreased chances of fall more than routine exercise. The results of the study were consistent with the community-based falls prevention program (25) and randomized controlled trials (10,26). PD patients develop dysrhythmic, slow, and shuffling gait (27). Loss of balance also occurs as disease advances (8). Tai Chi is a balance-based exercise. In this exercise, the center of gravity varies with each movement, challenging the balance control system (28). This exercise involves gentle and slow movements along with relaxation induced via deep breathing. Tai Chi has been reported to be very effective in preventing falls in elderly individuals (26). PD patients that perform Tai Chi experience body awareness and balance improvement (15). Tai Chi is recommended for PD patients who have experienced postural instability and are at an increased risk of falling.

### Clinical evaluations

In PD patients, loss in motor function occurs due to the death of neurons in the *substantia nigra*, which is a region of the central nervous system. Mechanisms behind the occurrence of PD are not well understood but it is believed that it is due to insufficient production of dopamine, which results in successive aggregation of protein and alpha-synuclein in Lewy bodies present in neurons (1). The efficacy of Tai Chi specifically in case of PD is explained by its role in normalizing the levels of neurotransmitters including dopamine and acetylcholine in various regions of the brain, i.e., cortex, basal ganglia, and motor cortex feedback loop. When Tai Chi is practiced daily, it promotes the development of various *de novo* neural pathways in a PD patient that results in fast response to posture challenges (10). Tai Chi exercise delayed the introduction of levodopa.

### Adverse effects

No adverse effect of the physical therapy was observed in the patients. All the exercises were safe for PD patients, except for dance, which disturbed the balance of 2 PD patients and they experienced a fall; however, no exercise-related injury occurred.

### Limitations

Although the study spanned over a short period of time, its results supported Tai Chi as an effective therapy for PD patients. This retrospective study indicated a direction for future studies that would involve post-therapy measurements. The other limitations of the study were that all participants agreed voluntarily and the results of the study might suffer from the placebo effect (29). Generalizability of results can also be a problem as Tai Chi is popular in China but not in other populations, resulting in implementation issues. The effect of exercise on other parameters related to PD like rigidity of skeletal muscles, depression, etc. was not evaluated. Duration of exercise time was relatively short. A randomized clinical trial is required for a longer period of exercise time.

### Conclusions

The results of this study supported that Tai Chi was an effective meditation technique for people who have mild to moderate Parkinson's disease. The incorporation of Tai Chi in the daily life of Parkinson's disease patients allowed them to stay functionally and physically active. Improvement of physical parameters indicated that Tai Chi had the potential to slow down the progression of Parkinson's disease and delay the introduction of levodopa.

## Supplementary Material

Click here to view [pdf].
